# XPO1 serves as a prognostic marker involving AKT/MAPK/TGFBR1 pathway in OSCC

**DOI:** 10.1002/cam4.70076

**Published:** 2024-08-23

**Authors:** Ying Han, Ying Peng, Haofeng Xiong, Liujun Zeng, Tianyi Zhang, Kun Xia, Xin Hu, Tong Su

**Affiliations:** ^1^ Department of Stomatology, Center of Stomatology, Xiangya Hospital Central South University Changsha Hunan China; ^2^ Research Center of Oral and Maxillofacial Tumor Xiangya hospital of Central South University Changsha Hunan China; ^3^ Institute of Oral Cancer and Precancerous Lesions Central South University Changsha Hunan China; ^4^ National Clinical Research Center for Geriatric Disorders (XiangYa Hospital) Changsha Hunan China; ^5^ Center for Medical Genetics & Hunan Key Laboratory of Medical Genetics, School of Life Sciences Central South University Changsha Hunan China

**Keywords:** bioinformatics analysis, OSCC, prognosis, XPO1

## Abstract

**Background:**

Exportin 1 (XPO1) is a nuclear export protein that facilitates the transportation of various substances. XPO1 promotes tumor development as a poor prognostic factor in a variety of tumors and is a therapeutic target for screening inhibitors. However, the role of XPO1 in oral squamous cell carcinoma (OSCC) has yet to be determined.

**Methods:**

The expression patterns of XPO1 mRNA in OSCC were investigated using bioinformatics tools, and the expression levels of XPO1 protein in OSCC specimens were confirmed by immunohistochemical assays. Survival analysis was conducted to evaluate the impact of XPO1 on prognosis. GO and KEGG enrichment analyses were utilized to uncover the signaling pathways mediated by XPO1. Additionally, we examined the association between XPO1 and AKT/MAPK/TGFBR1 and immune infiltration.

**Results:**

XPO1 mRNA and protein expression levels were significantly enhanced in OSCC and associated with OSCC severity. Enhanced XPO1 expression was indicative of poor survival. Functional analysis showed that XPO1 mediated pathways associated with cell cycle and DNA replication and reduced immune infiltration in OSCC. Additionally, XPO1 mRNA and protein expression levels had significant positive relationships with AKT/MAPK/TGFBR1.

**Conclusions:**

XPO1, as a marker of poor prognosis in OSCC, can promote OSCC through AKT/MAPK/TGFBR1.

## 1 INTRODUCTION

Oral cancer constitutes about 2% of all cancer cases, and oral squamous cell carcinoma (OSCC) represents about 90% of all oral cancer types worldwide.^1^ Despite the significant advances in the management of OSCC in recent decades, the 5‐year survival rate for OSCC patients remains below 60%.^2^ The main causes of death in OSCC patients are metastasis and recurrence. The mechanisms underlying the occurrence and development of OSCC are diverse and need to be elucidated in more detail. In developing countries, betel quid chewing, smoking, and alcohol consumption are common risk factors for OSCC development.^3^ Therefore, elucidating the molecular mechanism of OSCC occurrence and discovering new biomarkers for the diagnosis and treatment of OSCC has become an urgent task to explore new OSCC treatment strategies.

Transport of proteins across the nuclear envelope is critical for cellular homeostasis, and dysregulation of this fundamental process affects cellular processes such as inflammation, the cell cycle, and apoptosis.^4^ But this process is abnormal in the progression of tumors. Exportin 1 (XPO1), also known as Chromosomal region maintenance 1 (CRM1), is a protein transporter that facilitates the nucleocytoplasmic shuttling of most tumor suppressor proteins (TSPs) and growth regulators.^5^ XPO1 is upregulated in many malignancies, including pancreatic, ovarian, glioma, lung, gastric, prostate, and colorectal cancers, and is associated with worse prognosis. XPO1 mediates cell proliferation by maintaining nuclear and chromosomal structure, controlling mitogen and chromosomal segregation, and regulating the subcellular localization of oncogenes and TSPs which are NES‐containing.^4^ Therapies targeting aberrant XPO1 activation in tumors are entering the clinical trials stage. However, the function of XPO1 in OSCC remains unknown and warrants further investigation.

In this study, transcriptome data from public accessible databases was used to investigate the differential expression of XPO1 in OSCC samples and normal samples. Our own immunohistochemical microarray data was then used to validate these findings. Next, bioinformatics analysis was used to determine XPO1's biological, prognostic, and predictive roles in OSCC. Finally, the relationship between XPO1 and these biomarkers of proliferation, apoptosis, and immunity was explored.

## 2 MATERIALS AND METHODS

### 2.1 Acquisition and analysis of transcriptome data

The expression differences of XPO1 were investigated through the analysis of these two datasets by downloading GSE25099^6^ and GSE30784^7^ from the GEO website using R software and GEOquery package. There are 229 samples in GSE30784, of which 167 are samples of OSCC and 45 are samples of normal oral tissue. There are 79 samples in GSE25099, comprising 22 samples of normal oral tissue and 57 samples of OSCC. Download transcriptome data (HTSeq—Counts and HTSeq—FPKM files) and all clinical information related to patients with head and neck squamous cell carcinoma (HNSC) in TCGA from UCSC Xena (https://xenabrowser.net/datapages/). In the HNSC data, data lacking clinical information and repeated sample data were excluded, samples from Hypopharynx, Larynx, Lip, Oropharynx, and Tonsil were excluded, only the samples of oral tumors were retained. Finally, 360 samples were included in the study.

### 2.2 Study on the mRNA expression level of XPO1 and survival analysis between normal and OSCC tissues

To compare the mRNA expression levels of XPO1 in normal tissues and OSCC, transcriptome data from the GEO database GSE25099, GSE30784 dataset, and TCGA dataset were combined. Download the TCGA expression profile and follow‐up data, use “survminer” R packages to identify the optimal cut off point. We then split all OSCC patients into two groups for the analysis that follows: one for the high‐expression group of XPO1 and another for the low‐expression group. The basic R package is used to analyze the correlation of XPO1 expression with age, gender, TNM stage, pathological stage, prognosis, etc. In addition, we performed survival analysis to assess the prognostic significance of XPO1, and ROC analysis to test the diagnostic accuracy of XPO1 in discriminating normal and OSCC specimens.

### 2.3 Screening and enrichment analysis of genes associated with the XPO1 mRNA level

The edgeR package was applied to identify for differentially expressed genes (DEGs) with |logFC| >0.7 and *p*‐value <0.05.^8^ Use volcano plots to show DEGs. Functions potentially involved in XPO1 were annotated using Gene Set Enrichment Analysis (GSEA), Gene Ontology (GO), including biological process (BP), molecular function (MF), cellular component (CC) and KEGG via the clusterProfiler package.^9^, ^10^

### 2.4 Sample collection

The sample for this study was drawn from a previous cross‐sectional, descriptive and observational study by the subject group.^11^ Tissue samples of NOM, AT and OSCC were collected between May 2018 and May 2019 at the Center of Stomatology and the department of Oral and Maxillofacial Surgery, Xiangya Hospital, Central South University. OSCC patients and healthy volunteers were included or excluded based on specific criteria. The inclusion criteria for OSCC patients were: histological confirmation of OSCC, age range of 20–80 years, any gender, and standardized radical surgery of OSCC. The exclusion criteria for OSCC patients were: concurrent multiple cancer (except for metastatic OSCC) or previous cancer history before OSCC. The inclusion criteria for healthy volunteers were: age range of 20–80 years, any gender, and no history of smoking, drinking or betel nut chewing. The exclusion criteria for healthy volunteers were: other oral diseases or systemic diseases. The researchers collected OSCC tissues from the resected tumor tissues during the surgery without compromising the pathological diagnosis. The researchers obtained paired AT from the 2 cm‐extended margin of OSCC resection and verified the absence of OSCC pathologically, to minimize patient injury. They also collected clinicopathological parameters of OSCC patients simultaneously. The study used the 8th edition of the American Joint Committee on Cancer staging system for staging criteria and the 4th edition of the World Health Organization classification of head and neck tumors for histopathological classification. NOM tissues came from healthy volunteers.

### 2.5 Tissue microarray fabrication

All samples required for the experiment were fixed in 10% formalin. The area of tumor and epithelial tissue was determined by hematoxylin and eosin staining. The tissue cores were arranged on the blank recipient based on the design of the tissue chip spotter, and the tissue microarray was obtained using a slicer to perform serial slicing of. The tissue microarray of 17 NOM samples, 61 OSCC samples, and 47 paired AT samples was constructed in collaboration with Shanghai Biochip Company, Ltd. in Shanghai, China.

The experiments were conducted with the informed and written consent of each participant, following the World Medical Association Declaration of Helsinki (version 2002) and additional requirements. The study was reviewed and approved by the Ethics Committee of the School of Life Sciences at Central South University (No. 2020‐1‐42).

### 2.6 Immunohistochemical analysis

IHC was utilized to assess the protein expression of XPO1 (SANTA CRUZ, sc‐74,454, 1:200) in normal oral mucosa (NOM), oral potentially malignant disorders (OPMD), and OSCC tissues. The study evaluated the correlation between XPO1 and biomarkers of hypoxia, migration, proliferation, apoptosis, and immune cells. Details (antibody company, product number, dilution ratio) of part markers (HIF‐1α, E‐cadherin, Ki67, cleaved‐caspase3) have been described in our previous study. In addition to the above markers, PCNA (1:4000, 2586, Cell Signaling Technology), N‐cadherin (1:250, 13,116, Cell Signaling Technology), β‐catenin (1:100, 8480, Cell Signaling Technology), P53 (1:200, 48,818, Cell Signaling Technology), TGFBR1 (1:500, ab31013, Abcam), MAPK (1:400, 8690, Cell Signaling Technology), AKT (1:300, 4691, Cell Signaling Technology) were also employed in this study to explored the function of XOP1. The IHC protocol was presented in our previous study.^11^

### 2.7 Analyzing tissue microarrays and IHC quantitatively

The IHC images were scanned by the Servicebio company in Wuhan. The circled areas were analyzed by using Image‐Pro Plus software (version 6.0) to determine the integrated optical density (IOD) and areas of interest. We measured relative protein expression by comparing IOD and interested areas. The IHC was quantitatively assessed twice by independent researchers.

### 2.8 Ethics statements

The experiments were undertaken with the understanding and written consent of each subject and according to the World Medical Association Declaration of Helsinki (version 2002) and the additional requirements. Bioinformatics data in this study come from TCGA (https://tcga‐data.nci.nih.gov/tcga/tcga) and GEO (www.ncbi.nlm.nih.gov/geo) datasets. There are no ethical, licensing, or security concerns related to these data. All specimens were obtained with written informed consent signed by the patient. The histological research (IHC) in this study has been approved by the Ethics Committee of the School of Life Sciences, Central South University (NO.2020–1–42).

### 2.9 Immune cell infiltration and putative drugs identification

The association of XPO1 with immune infiltration was analyzed using the single‐sample gene set enrichment analysis (ssGSEA) algorithm in the GSVA package.^12^, ^13^ The estimate package is used to evaluate the relationship between XPO1 and stromalscore, immunescore, and ESTIMATEScore.^14^

### 2.10 Drug sensitivity analysis

We obtained the expression data of XPO1 from the CellMiner database for the nci‐60 cell line drug panel. We then computed the Pearson correlation coefficient between XPO1 and 792 FDA‐approved drugs undergoing clinical trials. Finally, we filtered and visualized the results (*p* < 0.05).^15^

### 2.11 Statistical analysis

The quantitative data of immunohistochemistry were tested for normality before statistical analysis. T‐tests were applied to data that followed a normal distribution and nonparametric tests to data that did not. To investigate the survival and prognostic factors of OSCC patients, we used both Kaplan–Meier and Cox regression analyses. We used univariate Cox regression to identify prognostic factors. Pearson correlation analysis assessed the associations between two variables. A two‐tailed *p‐value* of less than 0.05 was considered as significant. We performed statistical analysis and visualization of results using R software (version 4.1.3) and its packages.

## 3 RESULTS

### 3.1 Comparison of XPO1 between normal tissues and OSCC and its clinicopathological features

**FIGURE 1:**
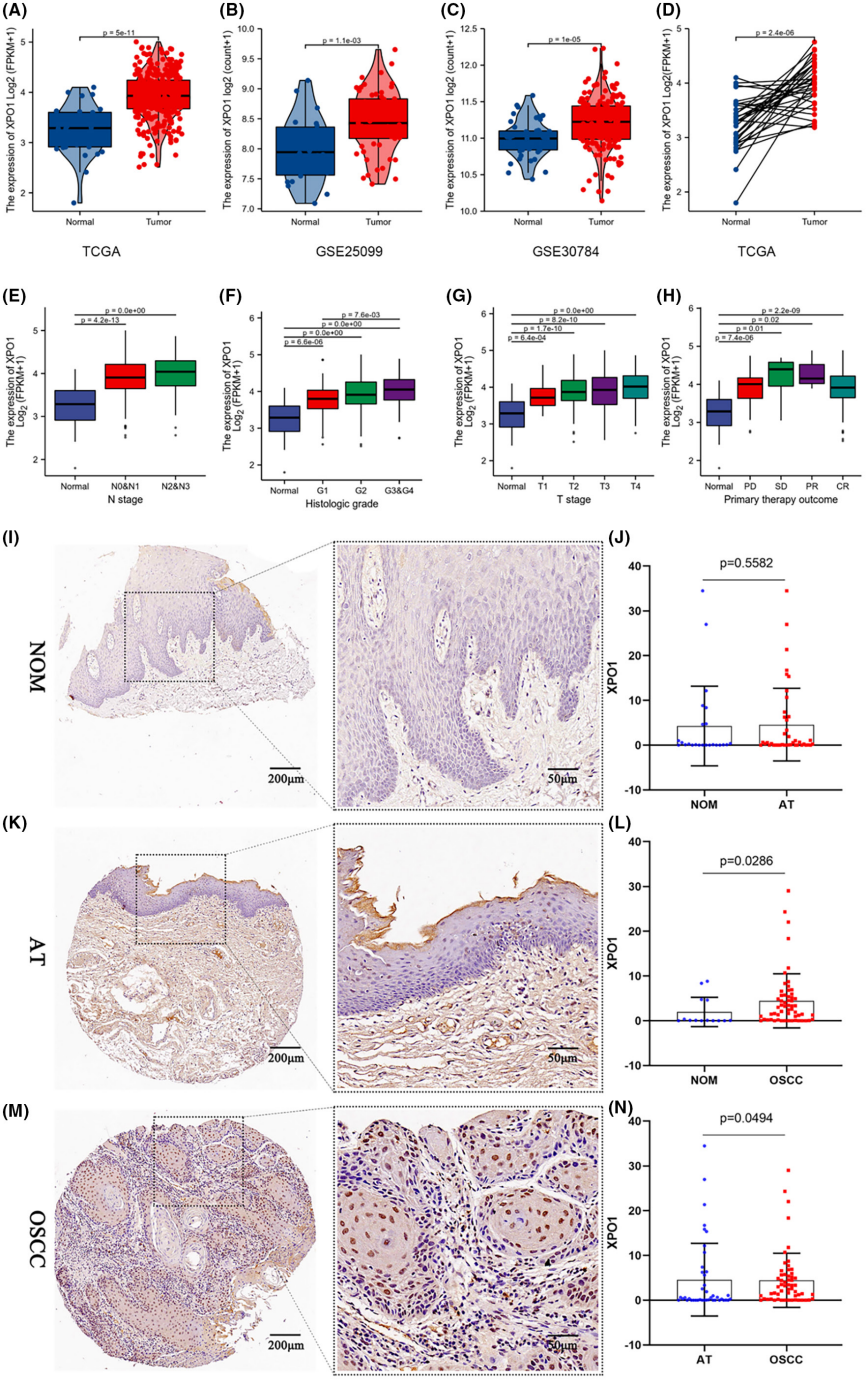
Comparison of XPO1 between normal tissue and OSCC tissue and its relationship with clinicopathological features. Immunohistochemical and quantitative comparison of XPO1 in normal, adjacent tissues and OSCC. (A) unpaired comparison of the mRNA expression levels of XPO1 in tumor and normal samples in TCGA‐OSCC. (B, C) unpaired comparison verification the mRNA expression levels of XPO1 in tumor and normal samples in GEO25099 and GSE30784. (D) Pairwise comparison of the mRNA expression levels of XPO1 in tumor and normal samples in TCGA‐OSCC. (E–G) The expression levels of XPO1 in different N stage, Histologic grade and T stage in OSCC samples were compared. (H) the expression level of XPO1 comparing different treatment outcomes of OSCC patients. (I). IHC images of XPO1 in normal oral tissue (NOM). (K). IHC images of XPO1 in paired adjacent tissues (AT) from OSCC (M). IHC images of XPO1 in OSCC. (J) Unpaired Wilcoxon test comparing the levels of XPO1 protein in AT and NOM. (L) Unpaired Wilcoxon test comparing the levels of XPO1 protein in OSCC and NOM. (N) Unpaired Wilcoxon test comparing the levels of XPO1 protein in OSCC and AT.

Using bioinformatics analysis, we compared the levels of XPO1 mRNA expression in OSCC and oral normal tissues. The expression of XPO1 was compared between tumor and normal samples in the OSCC dataset of TCGA. The results indicate that XPO1 mRNA levels were significantly upregulated in tumor samples compared to normal samples (Figure 1A), which was consistent with the paired results (Figure 1D). To confirm these findings, we acquired two datasets of OSCC from the GEO database: GSE25099 and GSE30784. Our analysis yielded results consistent with those of TCGA‐OSCC, indicating a significantly higher mRNA level of XPO1 in OSCC compared to normal tissues (see Figure 1B,C). Furthermore, we acquired clinical data from the TCGA‐OSCC dataset samples and examined the correlation between XPO1 and clinical information. The study findings indicate that the mRNA expression of XPO1 was significantly higher in N2&N3 samples compared to N0&N1 (Figure 1E). Additionally, the expression of XPO1 increased progressively with the advancement of T stage and Histologic grade (Figure 1F,G), and decreased with disease remission (Figure 1H). After we collected the samples, we used immunohistochemistry to compare the expression levels of XPO1 in normal tissues, adjacent tissues and OSCC. The results showed that the expression of XPO1 was higher in OSCC than in adjacent tissues, and in adjacent tissues than in normal tissues (Figure 1I–N).

### 3.2 Prognostic and functional analysis of XPO1 in OSCC

**FIGURE 2:**
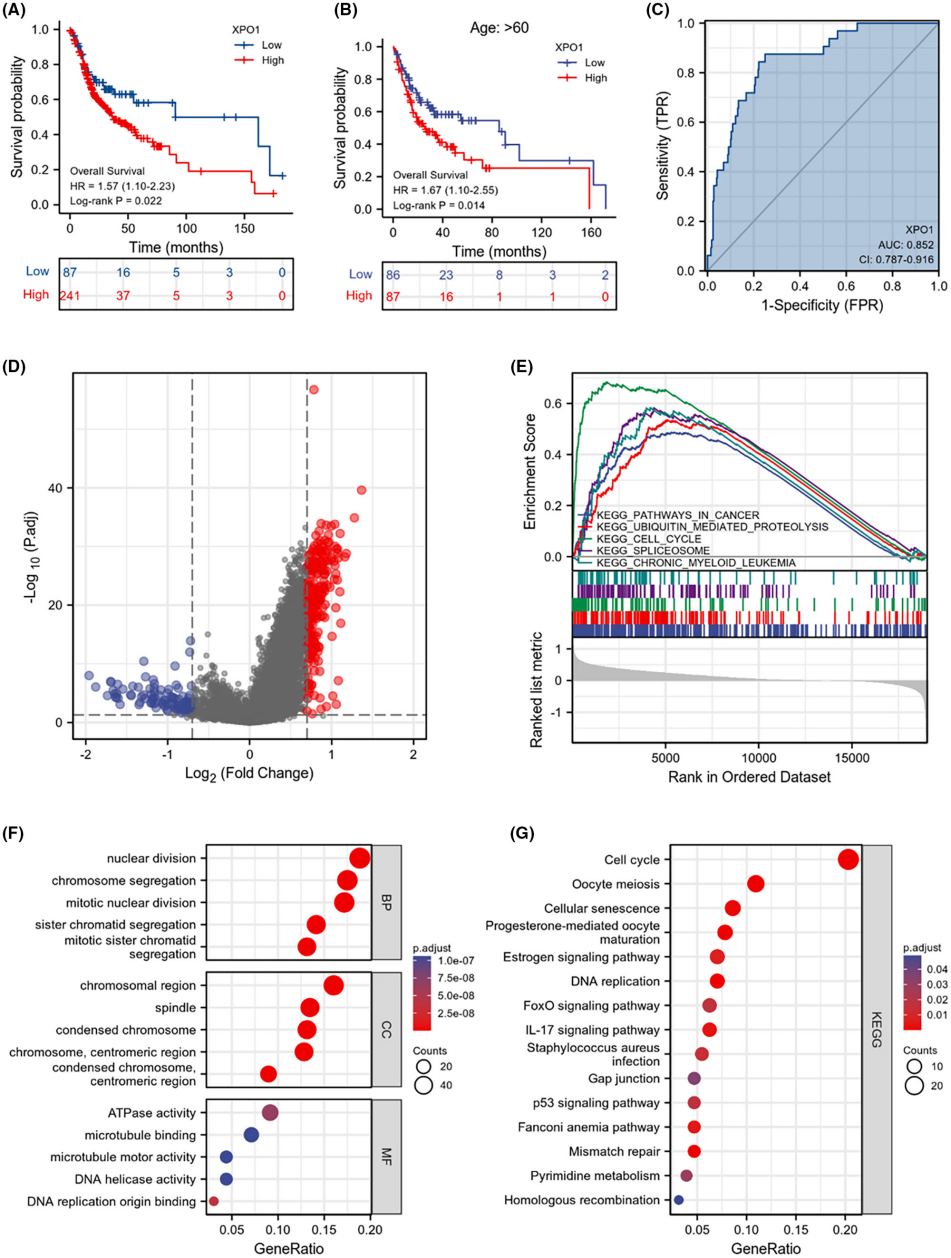
(A) Overall survival analysis in OSCC between groups with high and low XPO1 mRNA levels. (B) Overall survival analysis between high‐level and low‐level groups of XPO1 mRNA in patients over 60 years of age in OSCC. (C) ROC analysis evaluated the value of XPO1 as an indicator for distinguishing normal samples from OSCC samples. (D) The volcanic plot of differentially expressed genes (DEGs) was screened by groups of XPO1 mRNA at high and low levels. (E) GSEA was performed to explore differences in signaling pathways between low‐ and high‐expressing XPO1 group datasets in OSCC. (F, G) GO and KEGG analysis of XPO1‐related signatures in OSCC.

Three hundred and twenty eight samples were split into two groups by the TCGA database according to the optimal degree of separation. Of these, 241 were patients with high XPO1 mRNA levels, and 87 were patients with low XPO1 mRNA levels. Survival analysis indicated that patients with high XPO1 mRNA levels had a worse prognosis than those with low XPO1 mRNA levels (Figure 2A). Among them, 173 patients over the age of 60 were separated into two groups based on their XPO1 expression levels. The prognosis was statistically significant (Figure 2B). The ROC curve showed (Figure 2C) that XPO1 in OSCC could be used to distinguish normal samples from tumor samples. In order to further determine the function of XPO1 in OSCC, we conducted survival analysis on 329 samples from TCGA‐OSCC. After excluding samples without survival data, we divided the remaining samples into two groups: 241 cases with high expression of XPO1 and 88 cases with low expression of XPO1. A total of 1650 DEGs were identified, with 1000 genes upregulated and 650 genes downregulated. The volcano plot in Figure 2D displays the p‐values and fold changes for all DEGs. GSEA, GO and KEGG analyses were conducted for the significantly differential genes associated with XPO1 in OSCC. Results from GSEA showed that XPO1 activated KEGG Pathways in cancer, KEGG_Ubiquitin mediated proteolysis, KEGG_Cell cycle, KEGG_Spliceosome, and KEGG_Chronic myeloid leukemia (Figure 2E). GO analysis revealed that the BP of significantly different genes related to XPO1 was primarily involved in mitotic nuclear division, chromosome segregation, mitotic sister chromatid segregation, nuclear division, sister chromatid segregation, MF involves DNA replication origin binding, ATPase activity, DNA helicase activity, microtubule binding, microtubule motor activity, CC mainly focused on the chromosomal region, chromosome centromeric region, condensed chromosome, condensed chromosome centromeric region, spindle(Figure 2F). KEGG analysis showed that the signaling pathways of significantly different genes related to XPO1 mainly involved Cell cycle, DNA replication, IL‐17 signaling pathway, p53 signaling pathway, and FoxO signaling pathway (Figure 2G).

### 3.3 Association between immune infiltration and XPO1

**FIGURE 3:**
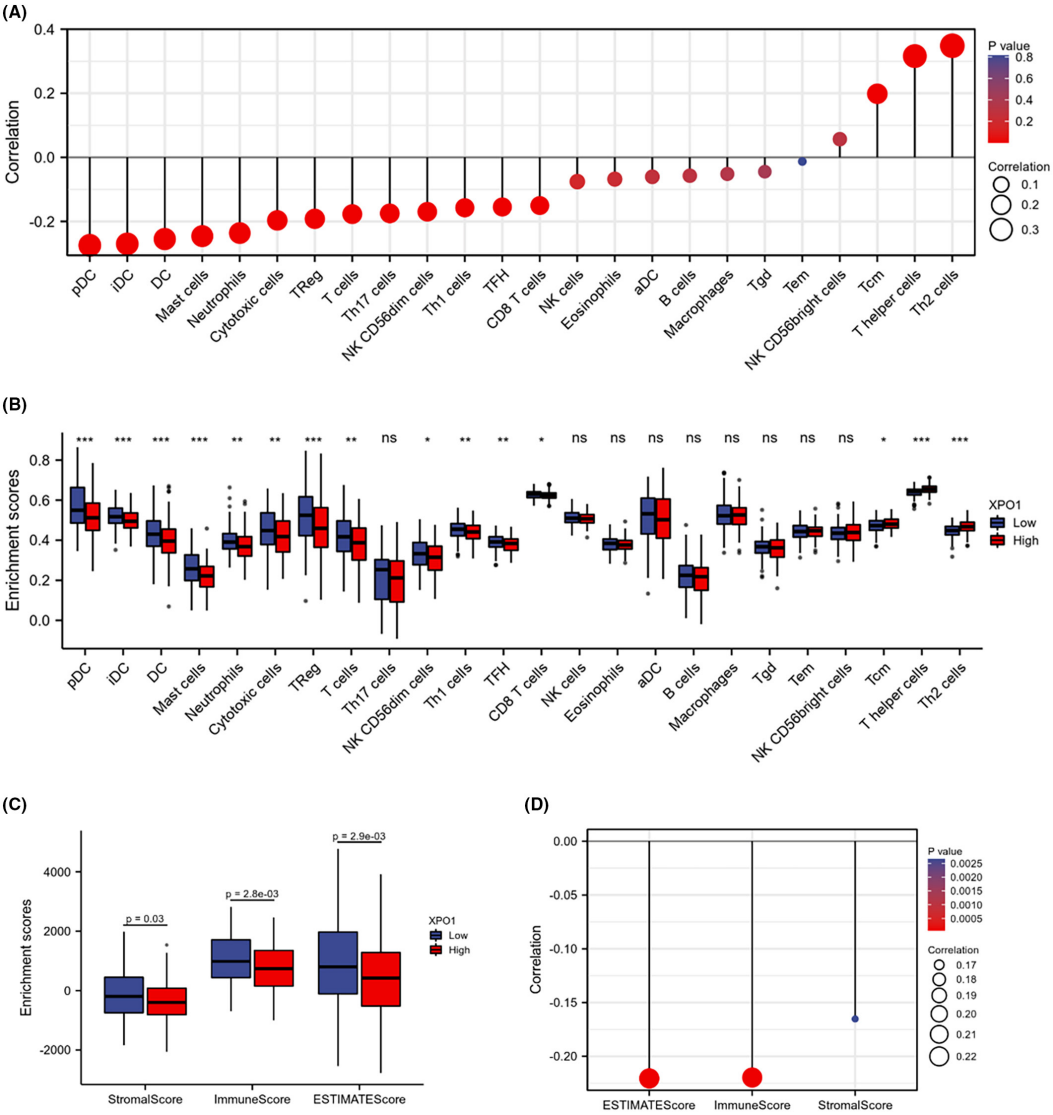
Associations of XPO1 expression to immune infiltration. (A) The correlations of XPO1 expression and immune infiltration in OSCC. (B) Comparison of enrichment scores between high and low expression of XPO1 in 24 immune cells in OSCC. (C) Comparison of enrichment scores of stromalscore, immunescore and ESTIMATEScore between high and low XPO1 expression in OSCC. (D) The correlation between XPO1 and ESTIMATE score, immune score, stromal score in OSCC. *p < 0.05.; **p < 0.01; ***, *p* < 0.001; ****p < 0.0001.

Based on immune cell marker gene lists and gene expression profiles, ssGSEA identified 24 immune cell infiltrates. The mRNA levels of XPO1 in OSCC tissues showed a moderate positive correlation with the levels of Th2 cells, T helper cells, and Tcm, with correlation coefficients greater than 0.2 (Figure 3A). Comparison of immune infiltration the immune infiltration of 24 immune cells in the high and low XPO1 mRNA level groups showed that in addition to “Th17 cells”, “NK cells”, “Eosinophils”, “aDC”, “B cell”, “Macrophages”, “Tgd”, “Tem”, and “NK CD56 bright cells”, the others were all statistically significant (Figure 3B). The stromalscore, immunoscore and ESTIMATEScore enrichment scores of high XPO1 expression in OSCC were lower than those of XPO1 low expression group (Figure 3C). Correlations between XPO1 and ESTIMATE scores, immune scores, and stromal scores in OSCC were negatively correlated (Figure 3D). This indicated that highly expressed XPO1 suppressed immune infiltration in OSCC and promoted OSCC progression.

### 3.4 Association of XPO1 with biomarkers of proliferation and apoptosis

**FIGURE 4:**
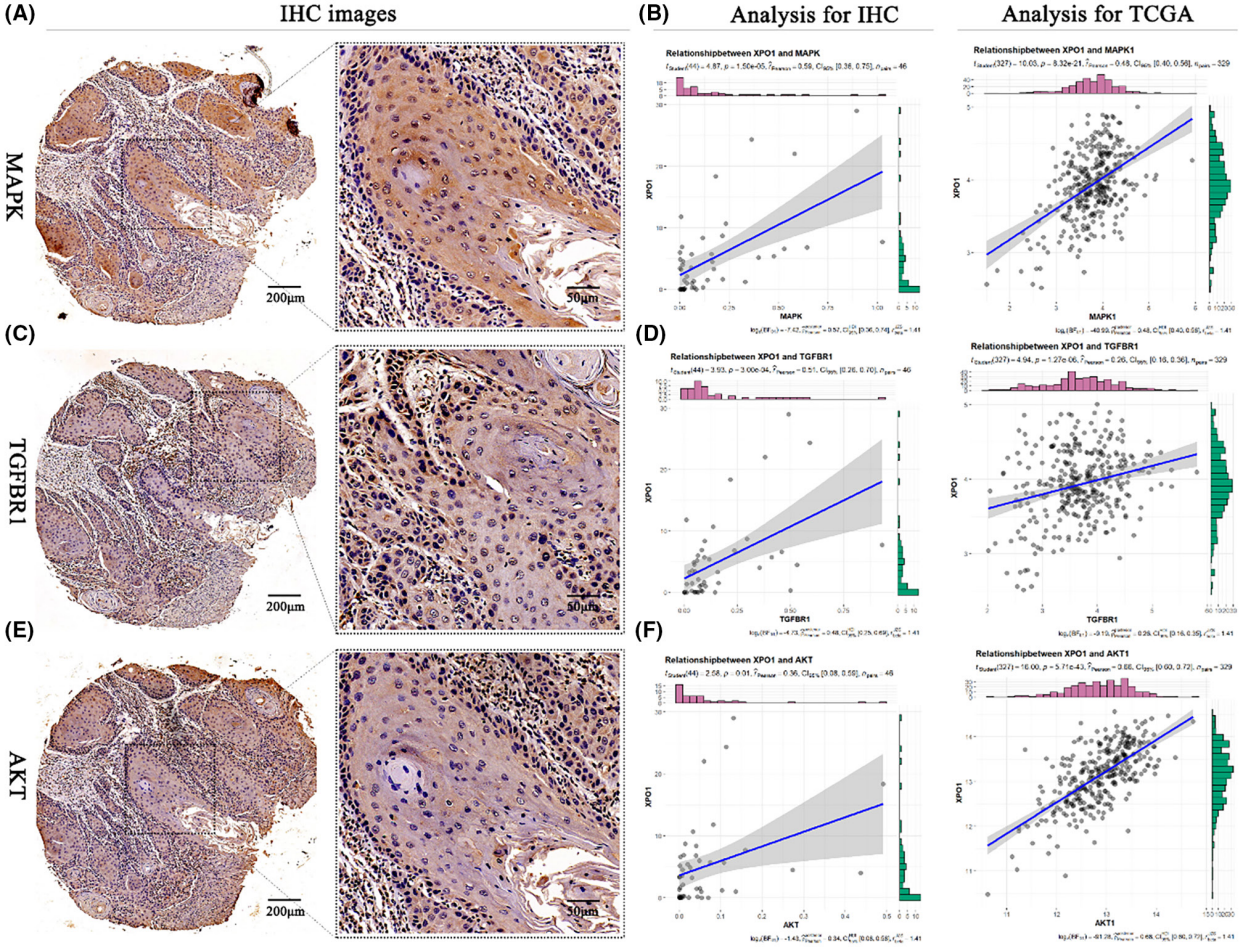
IHC images of AKT/MAPK/TGFBR1 and its correlation with XPO1 in OSCC tissues. (A) IHC images of MAPK. (B) Correlation of MAPK with XPO1 at protein and mRNA levels. (C) IHC images of TGFBR1. (D) Correlation of TGFBR1 with XPO1 at protein and mRNA levels. (E) IHC images of AKT. (F) Correlation of TGFBR1 with XPO1 at protein and mRNA levels.

**FIGURE 5:**
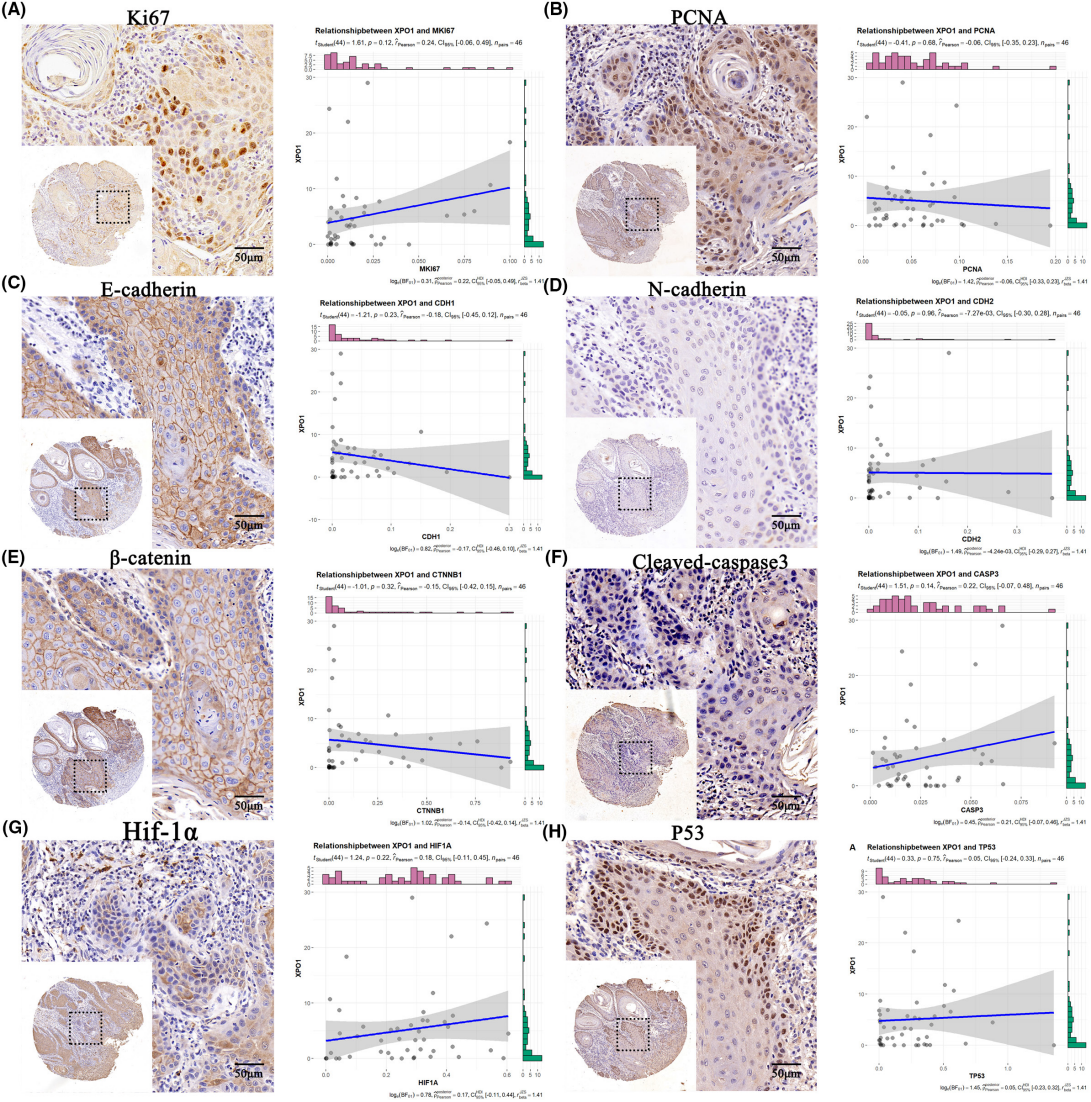
IHC images of proliferation and apoptosis biomarkers and their correlation with XPO1 at the protein level. (A) Ki67. (B) PCNA. (C) E‐cadherin. (D) N‐cadherin. (E) β‐catenin. (F) Cleaved‐caspase3. (G) Hif‐1α. (H) P53.

Functional analysis of XPO1 showed that XPO1 was mainly associated with cell cycle and DNA replication, so we analyzed the correlation of XPO1 with key genes implicated in cell cycle and DNA replication, as shown in Figure 4A–F showing that MAPK1, TGFBR1, AKT1 are high in OSCC expression, and showed a strong positive correlation with XPO1 protein expression. (MAPK1, *n* = 46, *r* = 0.59, *p* = 1.50e‐05; TGFBR1, *n* = 46, *r* = 0.51, *p* = 3.00e‐04; AKT1, *n* = 46, *r* = 0.36, *p* = 0.01). Likewise, there was a positive correlation observed between the mRNA levels of AKT1, TGFBR1, MAPK1, and XPO1 in TCGA. The OSCC tissues did not exhibit any significant correlation between XPO1 and any of the following: PCNA, E‐cadherin, N‐cadherin, catenin, Cleaved‐caspase3, Hif‐1α, and P53 (Figure 5A–H). This implies that XPO1 may function through MAPK1, AKT1, and TGFBR1 to contribute to the occurrence and development of OSCC.

### 3.5 Drug screening against XPO1

**FIGURE 6:**
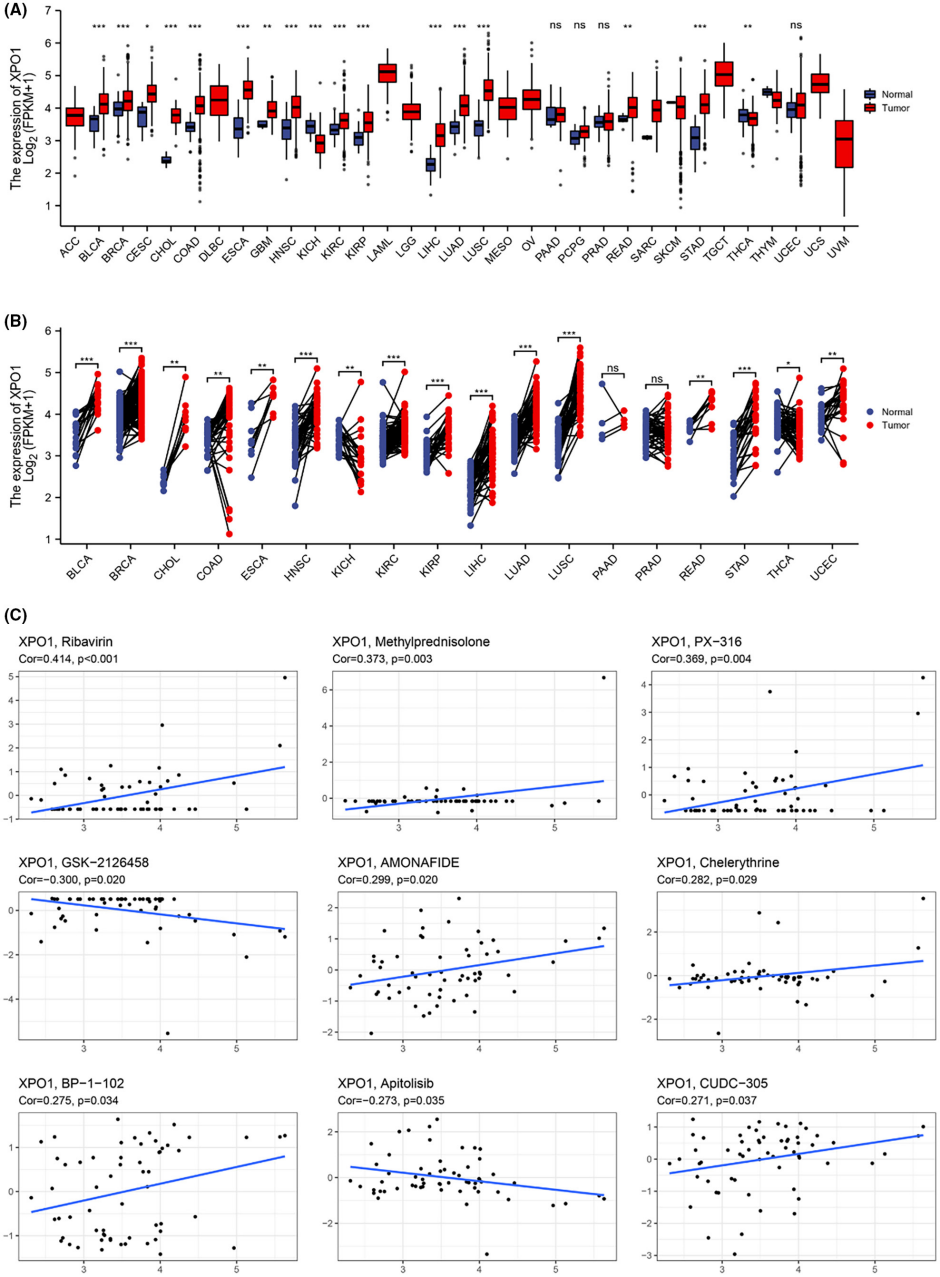
XPO1 expression levels in pan‐cancer. (A) Unpaired comparisons of the levels of XPO1 expression levels between tumor tissues from TCGA database and normal tissues from TCGA database (*p < 0.05, **p < 0.01, ***p < 0.001, ns = no significance). (B) Paired comparisons of XPO1 expression levels between tumor tissues from TCGA database and normal tissues from TCGA database (*p < 0.05, **p < 0.01, ***p < 0.001, ns = no significance). (C) Drug Sensitivity Analysis of XPO1.

Reported studies have shown increased expression of XPO1 in various types of cancer, including pancreatic, ovarian, glioma, lung, gastric, prostate, liver, cervical, multiple myeloid, leukemia, and colorectal cancers. This outcome is in line with our investigation of XPO1 expression in pan‐cancer, which is depicted in Figure 6A,B. XPO1 was significantly upregulated in BLCA, BRCA, CESC, CHOL, COAD, ESCA, GBM, HNSC, KIRC, KIRP, LIHC, LUAD, LUSC, READ, and STAD. These findings imply that XPO1 expresses abnormally in a variety of tumors and is a potential factor for tumor induction and progression. For this reason, XPO1 can serve as a target for diagnosing and treating OSCC, as well as pan‐cancer. Therefore, screening specific inhibitors against XPO1 can be used as broad‐spectrum anti‐cancer drugs. We used the CELLMINER database to conduct drug sensitivity experiments against XPO1 in various cells, and found that the expression of XPO1 was downregulated with increasing concentrations of GSK‐2126458 and Apotolisib, indicating that GSK‐2126458 and Apotolisib can be candidate XPO1 inhibitors for tumors However, unfortunately, Ribavirin, Methyprednisolone, PX‐316, AMONAFIDE, Chelerythyrine, BP‐1‐102, Cudc‐305 are positively correlated with XPO1, which also confirms a Chinese proverb that “All medicines have their own sideffects.”

## 4 DISCUSSION

Oral cancer is regarded as a serious threat to human health due to its high morbidity and mortality rate. The five‐year survival rate for oral cancer remains low due to delays in diagnosis. Therefore, for the global burden of oral cancer to be controlled, early detection, and prevention are essential.^16^ Finding valuable diagnostic and therapeutic biomarkers is particularly critical for the prevention and treatment of OSCC.

The primary mediators of macromolecule transport between the nucleus and cytoplasm are proteins belonging to the karyopherin‐β family. Exportin‐1 (XPO1) belongs to the nuclear transporter beta superfamily, also referred to as CRM1, is a protein and RNA transport mediator. XPO1 is upregulated in pancreatic, ovarian, glioma, lung, gastric, prostate, and colorectal cancers, and these conditions are also linked to a poor prognosis.^17^, ^18^, ^19^, ^20^, ^21^, ^22^, ^23^, ^24^ This is consistent with our analysis finding the upregulated expression of XPO1 in pan‐cancer. Additionally, our findings demonstrated that XPO1 was significantly upregulated in OSCC and was associated with poor prognosis.

There is compelling evidence that protein kinase B (AKT) contributes to cancer. Both normal and cancerous cells require the AKT1 protein to proliferate and survive.^25^ Research has indicated that AKT1 facilitates the occurrence and development of OSCC by regulating apoptosis, autophagy, invasion and migration, glycolysis, and angiogenesis. XPO1 promotes neuroblastoma cell proliferation through the PI3K/AKT pathway and activates cell cycle progression in G0/G1 phase by inhibiting p53 function.^26^ Additionally, our findings revealed that in OSCC, XPO1, and AKT1 had a positive correlation at the protein and mRNA levels. One of the pathways in human cancer that is most dysregulated is the mitogen‐activated protein kinase (MAPK) pathway.^27^ Numerous essential cellular processes, such as growth, senescence, and proliferation, are regulated by this pathway.^28^ OSCC cells undergo apoptosis when MAPK signaling is inhibited.^29^ Our findings also demonstrated that in OSCC, XPO1, and MAPK had a positive correlation at the protein and mRNA levels. One of the cellular signaling pathways in cancer that is most frequently altered is the transforming growth factor‐β (TGF‐β) pathway. Numerous facets of tumor biology, such as the regulation of the cell cycle, immunosuppression, apoptosis, angiogenesis, migration, and invasion, are regulated by TGF‐beta signaling.^30^ TGFBR1 is one of the main messengers of TGF‐β signaling.^30^ TGFBR1 promotes OSCC through proliferation, migration, invasion and angiogenesis.^31^ Additionally, XPO1 and TGFBR1 had a positive correlation at the protein and mRNA levels in OSCC, according to our data. After analyzing the function of XPO1, we discovered that the P53 signaling pathway, DNA replication, and cell cycle accounted for the majority of the significantly different XPO1‐related genes. Therefore, XPO1 promotes OSCC progression through AKT1/MAPK1/TGFBR1. In addition, XPO1 may activate Th2 cells through FoxO signaling pathway, IL17 signaling pathway, and inhibit the activation of DC cells, thereby inhibiting immune infiltration in OSCC.

XPO1 maintains cellular homeostasis by regulating the export of various cargoes from the nucleus to the cytoplasm, such as proteins and various RNA types.^32^ Accumulating evidence suggests that an important indicator of the occurrence of cancer and drug resistance is the export of drug targets, TSPs, and cell cycle regulatory proteins from the nucleus into the cytoplasm. As a result, XPO1 inhibitors block the nuclear export activity of particular proteins that XPO1 mediates, which reinstates the apoptotic pathway and increases the susceptibility of tumor cells to chemotherapy medications such as doxorubicin, tyrosine kinase inhibitor imatinib, etc. This specific inhibitor has emerged as a new focal point in cancer therapy. Numerous selective nuclear export inhibitors have been developed over time. In the absence of p53 mutation, Selinexor can effectively target DLBCL deteriorated by abnormal XPO1 expression.^33^ We found that XPO1 was significantly inhibited by GSK‐2126458 and Apitolisib through drug sensitivity experiments, indicating that these two drugs can be used as candidate inhibitors of XPO1 for the treatment of OSCC.

Although we found that XPO1 is abnormally upregulated in OSCC through public databases and self‐owned data, there are still many areas for further research in this study. First, additional samples must be obtained in order to confirm the prevalence of XPO1's aberrantly elevated expression in OSCC. Second, the regulatory mechanism of abnormally upregulated expression of XPO1 also needs to be elucidated. Third, our results show that XPO1 may regulate the cell cycle through AKT/MAPK/TGFBR1, and the DNA replication signaling pathway promotes the progression of OSCC, but the detailed mechanism needs to be further explored.

## 5 CONCLUSION

XPO1, as a poor prognostic biomarker in OSCC, can promote OSCC through AKT/MAPK/TGFBR1.

## AUTHOR CONTRIBUTIONS

**Ying Han:** Data curation (lead); formal analysis (lead); investigation (lead); methodology (lead); writing – original draft (lead). **Ying Peng:** Data curation (equal); investigation (equal); methodology (equal); writing – original draft (equal). **Haofeng Xiong:** Resources (supporting); software (supporting); validation (supporting); writing – review and editing (supporting). **Liujun Zeng:** Methodology (supporting); resources (supporting); software (supporting); validation (supporting); writing – review and editing (supporting). **Tianyi Zhang:** Formal analysis (supporting); resources (supporting); supervision (supporting); visualization (supporting); writing – review and editing (supporting). **Kun Xia:** Project administration (supporting); writing – review and editing (supporting). **Xin Hu:** Conceptualization (lead); funding acquisition (lead); project administration (equal); writing – original draft (supporting); writing – review and editing (supporting). **Tong Su:** Conceptualization (lead); funding acquisition (lead); writing – review and editing (equal).

## CONFLICT OF INTEREST STATEMENT

There is no conflict of interest.

## Data Availability

The original contributions presented in the study are included in the article material. Further inquiries can be directed to the corresponding author.

## References

[1] Lu Y , Zheng Z , Yuan Y , et al. The emerging role of exosomes in oral squamous cell carcinoma. Front Cell Dev Biol. 2021;9:628103. 33718365 10.3389/fcell.2021.628103 PMC7951141

[2] Ling Z , Cheng B , Tao X . Epithelial‐to‐mesenchymal transition in oral squamous cell carcinoma: challenges and opportunities. Int J Cancer. 2021;148(7):1548‐1561. 33091960 10.1002/ijc.33352

[3] Li WC , Lee PL , Chou IC , Chang WJ , Lin SC , Chang KW . Molecular and cellular cues of diet‐associated oral carcinogenesis—with an emphasis on areca‐nut‐induced oral cancer development. J Oral Pathol Med. 2015;44(3):167‐177. 24527773 10.1111/jop.12171

[4] Gravina GL , Senapedis W , McCauley D , Baloglu E , Shacham S , Festuccia C . Nucleo‐cytoplasmic transport as a therapeutic target of cancer. J Hematol Oncol. 2014;7:85. 25476752 10.1186/s13045-014-0085-1 PMC4272779

[5] Wang AY , Liu H . The past, present, and future of CRM1/XPO1 inhibitors. Stem Cell Investig. 2019;6:6. 10.21037/sci.2019.02.03 PMC6414360 30976603

[6] Peng CH , Liao CT , Peng SC , et al. A novel molecular signature identified by systems genetics approach predicts prognosis in oral squamous cell carcinoma. PLoS One. 2011;6(8):e23452. 21853135 10.1371/journal.pone.0023452 PMC3154947

[7] Chen C , Méndez E , Houck J , et al. Gene expression profiling identifies genes predictive of oral squamous cell carcinoma. Cancer Epidemiol Biomarkers Prev. 2008;17(8):2152‐2162. 18669583 10.1158/1055-9965.EPI-07-2893 PMC2575803

[8] Robinson MD , McCarthy DJ , Smyth GK . edgeR: a Bioconductor package for differential expression analysis of digital gene expression data. Bioinformatics. 2010;26(1):139‐140. 19910308 10.1093/bioinformatics/btp616 PMC2796818

[9] Yu G , Wang LG , Han Y , He QY . clusterProfiler: an R package for comparing biological themes among gene clusters. OMICS. 2012;16(5):284‐287. 22455463 10.1089/omi.2011.0118 PMC3339379

[10] Powers RK , Goodspeed A , Pielke‐Lombardo H , Tan AC , Costello JC . GSEA‐InContext: identifying novel and common patterns in expression experiments. Bioinformatics. 2018;34(13):i555‐i564. 29950010 10.1093/bioinformatics/bty271 PMC6022535

[11] Hu X , Xia K , Xiong H , Su T . G3BP1 may serve as a potential biomarker of proliferation, apoptosis, and prognosis in oral squamous cell carcinoma. J Oral Pathol Med. 2021;50(10):995‐1004. 33987877 10.1111/jop.13199

[12] Hänzelmann S , Castelo R , Guinney J . GSVA: gene set variation analysis for microarray and RNA‐seq data. BMC Bioinformatics. 2013;14:7. 23323831 10.1186/1471-2105-14-7 PMC3618321

[13] Bindea G , Mlecnik B , Tosolini M , et al. Spatiotemporal dynamics of intratumoral immune cells reveal the immune landscape in human cancer. Immunity. 2013;39(4):782‐795. 24138885 10.1016/j.immuni.2013.10.003

[14] Yoshihara K , Shahmoradgoli M , Martínez E , et al. Inferring tumour purity and stromal and immune cell admixture from expression data. Nat Commun. 2013;4:2612. 24113773 10.1038/ncomms3612 PMC3826632

[15] Luna A , Elloumi F , Varma S , et al. CellMiner cross‐database (CellMinerCDB) version 1.2: exploration of patient‐derived cancer cell line pharmacogenomics. Nucleic Acids Res. 2021;49(D1):D1083‐d1093. 33196823 10.1093/nar/gkaa968 PMC7779001

[16] Jitender S , Sarika G , Varada HR , Omprakash Y , Mohsin K . Screening for oral cancer. J Exp Ther Oncol. 2016;11(4):303‐307. 27849341

[17] Saulino DM , Younes PS , Bailey JM , Younes M . CRM1/XPO1 expression in pancreatic adenocarcinoma correlates with survivin expression and the proliferative activity. Oncotarget. 2018;9(30):21289‐21295. 29765539 10.18632/oncotarget.25088 PMC5940369

[18] Chen Y , Camacho SC , Silvers TR , et al. Inhibition of the nuclear export receptor XPO1 as a therapeutic target for platinum‐resistant ovarian cancer. Clin Cancer Res. 2017;23(6):1552‐1563. 27649553 10.1158/1078-0432.CCR-16-1333

[19] Shen A , Wang Y , Zhao Y , Zou L , Sun L , Cheng C . Expression of CRM1 in human gliomas and its significance in p27 expression and clinical prognosis. Neurosurgery. 2009;65(1):153‐160. 19574837 10.1227/01.NEU.0000348550.47441.4B

[20] Gupta A , Saltarski JM , White MA , Scaglioni PP , Gerber DE . Therapeutic targeting of nuclear export inhibition in lung cancer. J Thorac Oncol. 2017;12(9):1446‐1450. 28647672 10.1016/j.jtho.2017.06.013 PMC5572747

[21] Subhash VV , Yeo MS , Wang L , et al. Anti‐tumor efficacy of Selinexor (KPT‐330) in gastric cancer is dependent on nuclear accumulation of p53 tumor suppressor. Sci Rep. 2018;8(1):12248. 30115935 10.1038/s41598-018-30686-1 PMC6095850

[22] Gravina GL , Mancini A , Sanita P , et al. KPT‐330, a potent and selective exportin‐1 (XPO‐1) inhibitor, shows antitumor effects modulating the expression of cyclin D1 and survivin [corrected] in prostate cancer models. BMC Cancer. 2015;15:941. 26620414 10.1186/s12885-015-1936-z PMC4666032

[23] Aladhraei M , Kassem Al‐Thobhani A , Poungvarin N , Suwannalert P . Association of XPO1 overexpression with NF‐κB and Ki67 in colorectal cancer. Asian Pac J Cancer Prev. 2019;20(12):3747‐3754. 31870117 10.31557/APJCP.2019.20.12.3747 PMC7173379

[24] Saenz‐Ponce N , Pillay R , de Long LM , et al. Targeting the XPO1‐dependent nuclear export of E2F7 reverses anthracycline resistance in head and neck squamous cell carcinomas. Sci Transl Med. 2018;10(447):eaar7223. 29950445 10.1126/scitranslmed.aar7223

[25] Harsha C , Banik K , Ang HL , et al. Targeting AKT/mTOR in Oral cancer: mechanisms and advances in clinical trials. Int J Mol Sci. 2020;21(9):3285. 32384682 10.3390/ijms21093285 PMC7246494

[26] Pan L , Cheng C , Duan P , Chen K , Wu Y , Wu Z . XPO1/CRM1 is a promising prognostic indicator for neuroblastoma and represented a therapeutic target by selective inhibitor verdinexor. J Exp Clin Cancer Res. 2021;40(1):255. 34384466 10.1186/s13046-021-02044-z PMC8359549

[27] Santarpia L , Lippman SM , El‐Naggar AK . Targeting the MAPK‐RAS‐RAF signaling pathway in cancer therapy. Expert Opin Ther Targets. 2012;16(1):103‐119. 22239440 10.1517/14728222.2011.645805 PMC3457779

[28] McCubrey JA , Steelman LS , Chappell WH , et al. Roles of the Raf/MEK/ERK pathway in cell growth, malignant transformation and drug resistance. Biochim Biophys Acta. 2007;1773(8):1263‐1284. 17126425 10.1016/j.bbamcr.2006.10.001 PMC2696318

[29] Wu H , Li L , Ai Z , Yin J , Chen L . Pristimerin induces apoptosis of oral squamous cell carcinoma cells via G(1) phase arrest and MAPK/Erk1/2 and Akt signaling inhibition. Oncol Lett. 2019;17(3):3017‐3025. 30854080 10.3892/ol.2019.9903 PMC6365977

[30] Moore‐Smith L , Pasche B . TGFBR1 signaling and breast cancer. J Mammary Gland Biol Neoplasia. 2011;16(2):89‐95. 21461994 10.1007/s10911-011-9216-2 PMC4753062

[31] Wang Y , Jia RZ , Diao S , He J , Jia L . miRNA‐101 targets TGF‐βR1 to retard the progression of Oral squamous cell carcinoma. Oncol Res. 2020;28(2):203‐212. 31831099 10.3727/096504019X15761480623959 PMC7851522

[32] Azmi AS , Uddin MH , Mohammad RM . The nuclear export protein XPO1‐from biology to targeted therapy. Nat Rev Clin Oncol. 2021;18(3):152‐169. 33173198 10.1038/s41571-020-00442-4

[33] Deng M , Zhang M , Xu‐Monette ZY , et al. XPO1 expression worsens the prognosis of unfavorable DLBCL that can be effectively targeted by selinexor in the absence of mutant p53. J Hematol Oncol. 2020;13(1):148. 33148342 10.1186/s13045-020-00982-3 PMC7641823

